# Organophosphate Triesters and Their Transformation Products in Sediments of Mangrove Wetlands in the Beibu Gulf, South China Sea

**DOI:** 10.3390/molecules29030736

**Published:** 2024-02-05

**Authors:** Li Zhang, Yongze Xing, Peng Zhang, Xin Luo, Zengyuan Niu

**Affiliations:** 1 Guangxi Key Laboratory of Beibu Gulf Marine Resources, Environment and Sustainable Development, Fourth Institute of Oceanography, Ministry of Natural Resources, Beihai 536000, China; zhangli@4io.org.cn; 2 Key Laboratory of Tropical Marine Ecosystem and Bioresource, Fourth Institute of Oceanography, Ministry of Natural Resources, Beihai 536000, China; 3 College of Chemistry and Environmental Science, Guangdong Ocean University, Zhanjiang 524088, China; zhangpeng@gdou.edu.cn; 4 Technology Center of Qingdao Customs District, Qingdao 266109, China; rossi612@hotmail.com (X.L.); zyniuqd@163.com (Z.N.)

**Keywords:** organophosphate triesters, transformation products, mangrove, sediment, ecological risk

## Abstract

As emerging pollutants, organophosphate esters (OPEs) have been reported in coastal environments worldwide. Nevertheless, information on the occurrence and ecological risks of OPEs, especially the related transformation products, in mangrove wetlands is scarce. For the first time, the coexistence and distribution of OP triesters and their transformation products in three mangrove wetlands in the Beibu Gulf were investigated using ultrasonication and solid-phase extraction, followed by UHPLC-MS/MS detection. The studied OPEs widely existed in all the sampling sites, with the total concentrations ranging from 6.43 ng/g dry weight (dw) to 39.96 ng/g dw and from 3.33 ng/g dw to 22.50 ng/g dw for the OP triesters and transformation products, respectively. Mangrove wetlands tend to retain more OPEs than the surrounding coastal environment. Pearson correlation analysis revealed that the TOC was not the sole factor in determining the OPEs’ distribution, and degradation was not the main source of the transformation products in mangrove sediments in the Beibu Gulf. The ecological risks of selected OPEs for different organisms were also assessed, revealing a medium to high risk posed by OP diesters to organisms. The levels or coexistence of OPEs and their metabolites in mangroves need constant monitoring, and more toxicity data should be further studied to assess the effect on normal aquatic organisms.

## 1. Introduction

Organophosphate esters (OPEs) have been commonly used as flame retardants in various industries, and sometimes also as plasticizers, antifoaming agents and lubricants [1]. With the ban on traditional brominated flame retardants, the production and usage of OPEs have continually increased during recent years [2,3]. Since OPEs are simply added into the materials instead of chemically bonded, they tend to release into the environment during the production and usage process easily [1,4,5]. Consequently, there is growing evidence of high levels of OPEs pollution in coastal environments worldwide [6,7,8]. Moreover, toxic effects of OPEs on humans and biota, such as neurotoxicity, carcinogenicity, endocrine disrupting activity, reproductive and developmental toxicity, have also been reported [5,9,10,11,12]. Studies on the pollution of OPEs in aquatic environments have attracted more and more interest in recent years [1,13].

According to previous reports, OP triesters cannot only be rapidly metabolized in humans and animals [14,15,16] but also can be degraded via other ways in the environment, such as microbial degradation [17], base-catalyzed hydrolysis and photodegradation [18,19,20]. Besides OP diesters, which have been identified as the major transformation products [21,22,23], recent in vitro and in vivo studies also revealed that the hydroxylated transformation products of OPEs (OH-OPEs) were even more significant. Taking tris (2-butoxyethyl) phosphate (TBOEP) as an example, the levels of bis(2-butoxyethyl) hydroxyethyl phosphate (BBOEHEP) and bis(2-butoxyethyl) hydroxyl-3-butoxyethyl phosphate (3-OH-TBOEP) metabolites were significantly higher than those of bis(2-butoxyethyl) phosphate (BBOEP) in human liver and fish [3,23]. The OP transformation products generated through all the above process could be potential sources and will eventually remain in the environment. Notably, some transformation products, such as diphenyl phosphate (DPHP), were found to be even more toxic than the parent compound, triphenyl phosphate (TPHP) [20]. However, to date, most reports on the transformation products of OP triesters mainly focused on biotic samples [21,24,25], while studies on the environmental matrices are scarce to see. In particular, the pollution of hydroxylated transformation products in the environment has not been reported so far.

Mangrove wetland is a unique coastal transitional ecosystem, which has important social and economic value, as well as ecological service functions [26]. For example, the direct photosynthetic products of mangroves provide local residents with firewood, charcoal, food, and other daily necessities and production resources. And the distinctive root systems and dense canopy of mangroves provide them with strong wind resistance and wave reduction capabilities, protecting coastal residents for production and livelihood security. Meanwhile, mangroves provide abundant organic matter, serving as nurseries for marine animals, making significant contributions to nearshore fisheries and biodiversity conservation. Nevertheless, as an important sink of various pollutants, heavy metals, polycyclic aromatic hydrocarbons (PAHs), and the persistent organic pollutants (POPs), such as organochlorine pesticides, polychlorinated biphenyls (PCBs) and polybrominated Biphenyls (PBDEs), have also been reported in mangrove sediments [26,27,28,29,30], plants and organisms (e.g., mollusks, bivalves and fish) [31,32]. Furthermore, Bayen et al. [31] revealed the biomagnification of PCBs, OCPs, and PBDEs in a food web composed of twenty-four biota species, including algae, worms, crustaceans, and fish, in two mangrove sites in Singapore, and thunder crabs (Myomenippe hardwicki) and fish exhibited the highest contaminant levels [31]. Research on the toxic effects of the above-mentioned pollutants on mangrove plants and organisms has been reported. For example, PAH was found to inhibit the transpiration rate, stomatal resistance, and growth of Avicennia marina seedlings [33]. Additionally, PAHs, OCPs, and PCBs affected the growth of *Periophthalmus argentilineatus* (mudskippers) and led to malformations (absence of the left eye) [34]. The BDE-209 reduced microbial diversity and inhibited the nitrification potential activity of microbes [35,36]. Endosulfan can impact the survival rate of *Gambusia puncticulata* (guppy) [37]. Therefore, research on the typical organic pollutants in mangrove ecosystems holds high economic and social value, while also being scientifically significant for the protection of human health. Nevertheless, besides PAHs and POPs, very little information is available on the accumulation of emerging contaminants in mangrove ecosystems. Very recently, Xie et al. [38] studied the pollution of OP triesters in both mangrove plants and animals in Qi’ao island, China, and most OP triesters showed bioaccumulation potential in mangrove animals, especially the isodecyl diphenyl phosphate was found to have biomagnification property [26]. However, no information on the occurrence and the ecological risk of OP transformation products in mangrove wetlands has been reported. Therefore, given the widespread presence of OPEs in the environment and their toxicity, further attention is urgently needed for the pollution study of organophosphate triesters, as well as their transformation products in mangrove ecosystems, to better understand their environmental behaviors and ecological risks.

The Beibu Gulf, located in the northwest of the South China Sea, is one of the most important natural fishing and mariculture zones in China [1,39]. The tropical and subtropical climate and abundant seagoing rivers leave the Beibu Gulf surrounded by vast mangroves, which account for over 37% of the total mangrove area in China [40,41]. In this study, surface sediments were collected from three mangrove wetlands in the Beibu Gulf and OPEs, including twelve OP triesters and seven related transformation products, were comprehensively analyzed. The aims were to (1) investigate the co-occurrence of selected OP triesters and transformation products in mangrove wetlands in the Beibu Gulf; (2) evaluate the relationships between OP triesters and related transformation products, as well as the factors influencing their spatial distribution in mangrove wetlands; and (3) provide initial insights into the ecological risks of OPEs in the mangrove wetlands. To the best of our knowledge, this is the first study that simultaneously reports the pollution status of both OP triesters and their transformation products in a mangrove ecosystem.

## 2. Results and Discussion

### 2.1. Occurrence of OPEs in Mangrove Sediment

The concentrations of the selected OP triesters and their transformation products in the surface sediments of the studied mangrove wetlands are shown in Table 1. Except for TEP and TPRP, the other OP triesters were detected in all the sediments. The total levels of the 12 target OP triesters (Σ_12_OP_tri_) at the different sampling sites were 6.43–39.96 ng/g dw, with a mean value of 14.10 ng/g dw. To date, the pollution of OPEs in the mangrove wetlands is very limited. The total concentration of OP triesters in the present study was lower than that of the mangrove sediments in the Pearl River Estuary (13.2~377.1 ng/g dw, mean 54.9 ng/g dw [26] and 23.5~187 ng/g dw, mean 62.0 ng/g dw [38]). The Pearl River Delta is the most economically developed region in China, and its surrounding area has a high level of industrialization and urbanization, thus leading to higher levels of OPEs than those of the Beibu Gulf.

**Figure 1:**
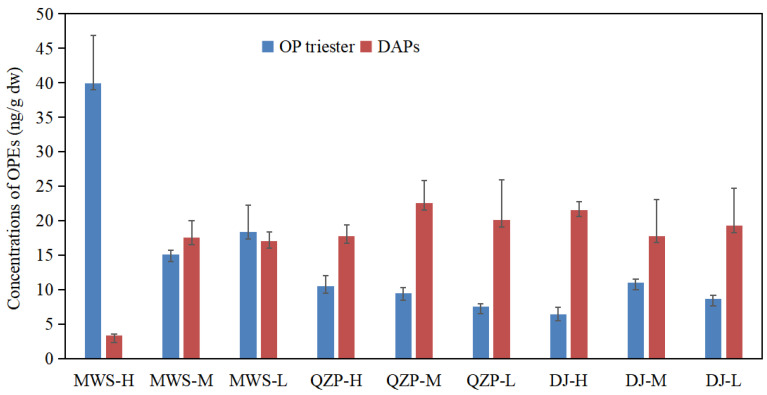
Occurrence and distribution of OP triesters and their degradation products in mangrove surface sediment in the Beibu Gulf.

The Σ_12_OP_tri_ concentration decreased in the order of MWS (73.43 ng/g dw) > QZP (27.41 ng/g dw) ≈ DJ (26.08 ng/g dw). The levels of the Σ_12_OP_tri_ concentration in the MWS were much higher than those in the other two mangrove areas. One explanation is that the surrounding abundant seagoing rivers, such as the Qin River, Maoling River and Dalan River, bring a high amount of OP triesters into the MWS mangrove, where they combine with the rich organic matter in the sediment and deposit. Moreover, there are intensive aquaculture areas around the MWS, thereby the pollution of OPEs in this area may also be associated with emissions from plastic products used during the aquaculture, as well as fishing processes. In contrast, the DJ and QZP mangroves are located in a rural area and a scenic area, respectively, and thus are less affected by human activities. Furthermore, when we compare the results of this work with our previous study on the Maowei Sea sediments, a higher Σ_12_OP_tri_ content in the MWS mangrove sediment was found. Considering mangroves are located in transitional zones between marine and land, this result also indicates that terrestrial input is a major source of OPE pollution in the coastal environment, and mangroves may play a significant role in retaining OPEs. Tam et al. [42] also found that the polycyclic aromatic hydrocarbon levels in mangrove sediment were significantly higher than those of surrounding marine bottom sediments. This phenomenon also can be inferred from the concentration distribution trend of the OP triesters along different intertidal zones, especially in the relatively heavily polluted MWS mangrove areas (Figure 1). As seen in Figure 1, there is a noticeable decrease in the Σ_12_OP_tri_ concentration in sediments from the high tide zone to the low tide zone, and the Σ_12_OP_tri_ level in the low tide zone is much closer to that in the surface sediment of the Maowei Sea reported by our previous study [1]. The decreasing trend is not clear in the DJ mangroves and QZP mangroves, which may be due to the lower Σ_12_OP_tri_ levels caused by the lower degree of terrestrial influence in these two areas.

A significant correlation between the Σ_12_OP_tri_ levels and TOC contents in the surface sediments was only found in the MWS mangrove area (r^2^ = 1.00, *p* = 0.018) (Table S1), indicating that the TOC was not the only factor determining the Σ_12_OP_tri_ levels. Variations in the relationships between the TOC and Σ_12_OP_tri_ levels among different mangrove forests were also reported by Hu et al. [26] and Tan et al. [43]. The above results revealed that the distribution of OP triesters in mangrove sediment may also be affected by other factors, such as compound degradation and the emission intensity.

For the first time, five OP diesters (DNBP, BBOEP, BCIPP, BDCIPP and DPHP) and two hydroxylated transformation products (the sum of which is designated as the Σ_7_OP_tp_) were analyzed in mangrove sediment in present study. And the concentration of the Σ_7_OP_tp_ ranged from 3.33 to 25.50 ng/g dw (mean 17.44 ng/g dw). Among which, BBOEHEP, BCIPP, DNBP and DPHP were detected in all the samples, followed by 3-OH-TBOEP (33%) and BBOEP (22%). Since no study on the transformation products in coastal sediment was reported, here the present result was compared with those from sewage sludge, soil and dust. The Σ_7_OP_tp_ in the present study was much lower than that of the sludge samples (17.0~1300 ng/g dw, mean 123 ng/g dw) [20] and indoor dust samples (up to 10^4^ ng/g dw) collected from Guangzhou [44,45]. Different from their parent compounds, the Σ_7_OP_tp_ concentration in the MWS mangrove (37.94 ng/g dw) was much lower than those in the DJ (58.46 ng/g dw) and QZP (60.38 ng/g dw) mangrove areas (Table 1). As for the concentration distribution along the different tidal zones, higher levels of Σ_7_OP_tp_ were found in the middle tidal zone, followed by the low tidal zone and high tidal zone for the MWS and QZP mangrove wetlands (Figure 1). The Σ_7_OP_tp_ concentration decreased in the order of the high tidal zone (21.57 ng/g dw) > the low tidal zone (19.29 ng/g dw) > the middle tidal zone (17.78 ng/g dw) in the DJ mangrove area, which is just contrary to that of the OP triesters. No significant correlation was found between the Σ_7_OP_tp_ levels and TOC contents in sediments in all three studied areas, revealing that the distribution of the OP transformation products was not determined by the TOC. Both anthropogenic activities and the degradation of the parent compounds could be possible influencing factors, which will be discussed in detail later. 

### 2.2. Composition Profiles of OPEs in Mangrove Sediments

**Figure 2:**
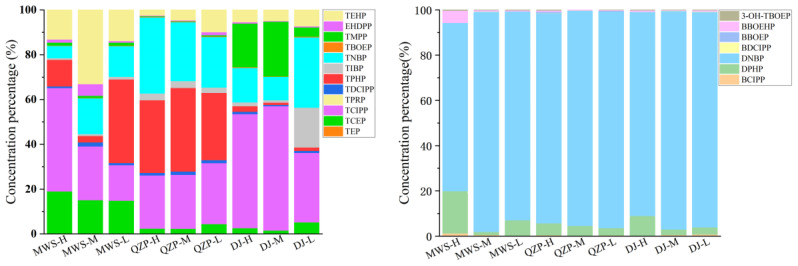
Concentration percentage of individual OPEs in surface sediment.

Overall, the total concentration of the main individual OP triesters detected in all the sites ranked as TCIPP (43.83 ng/g dw) > TPHP (21.61 ng/g dw) > TNBP (19.70 ng/g dw) > TEHP (15.76 ng/g dw) ≈ TCEP (14.13 ng/g dw), while the detailed compound composition varied among different areas (Figure 2). For the MWS mangrove, TCIPP was predominant, accounting for 34% of the Σ_12_OP_tri_ level in this area, followed by TCEP (17%), TEHP (17%) and TPHP (16%). For the QZP mangrove, TPHP accounted the highest concentration proportion of 33%, followed by TNBP (28%) and TCIPP (25%). Similar with the MWS mangrove, TCIPP accounted for most (46%) in the DJ mangrove area; however, TNBP and TMPP were secondly abundant compounds with proportions of 19% and 16%. TCIPP and TCEP are the two most frequently used OP triesters in industry as flame retardants [46] and were widely detected as major compounds in lots of coastal areas due to their large industrial production and persistent property in the environment [46,47,48,49]. The mangrove forests of MWS and DJ receive household and industrial wastewater discharge from the surrounding urban and rural areas, which leads to a high amount of TCIPP and TCEP. In the meantime, due to the mutagenicity and carcinogenicity of TCEP, it has been replaced by TCIPP gradually in recent years [46,50]. This could explain the result of TCIPP as the predominant OP triester in present study. TPHP is mainly used as flame retardants in electronic products, while TNBP is mainly used as plasticizers and lubricants [46]. Therefore, the household waste and shipping activities, especially in the QZP area, may be the main sources of TNBP and TPHP in the QZP and DJ mangrove forests. 

Different from the OP triesters, DNPB is the dominant transformation product among all three mangrove areas, accounting for more than 93% of the Σ_7_OP_tp_ concentration, followed by DPHP (~5%). Previous reports claimed that TNBP and TPHP tended to be degraded into related diesters via biodegradation in the environment [51,52]. To ascertain the relationship between DNBP and its parent TNBP, as well as between DPHP and its parent TPHP, in mangrove sediment, Pearson correlation analysis was performed. However, no significant correlation (*p* > 0.05) was observed, which indicated that the degradation of the parent compounds was not the main source of DNBP and DPHP in the studied area. As mentioned in Section 2.2, high levels of OP diesters were found in sludge samples and indoor dust samples (up to 10^4^ ng/g dw) [20,44,45]. In addition, DNBP and DPHP were noted as two industrialized compounds among all six studied OP diesters [20]. Therefore, external input may be an important reason for the high levels of DNBP and DPHP in the mangrove environment.

### 2.3. Ecological Risk Assessment

**Figure 3:**
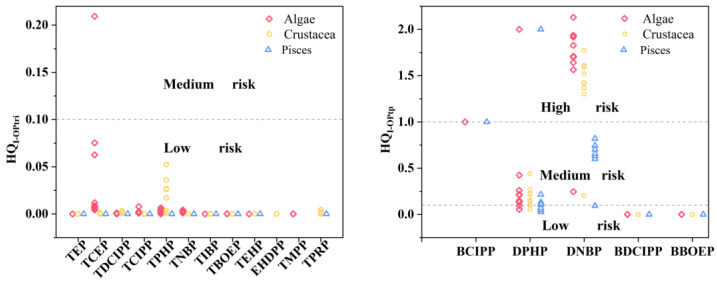
The ecological risk of individual OP triester (HQ_I-OPtri_) and transformation product (HQ_I-OPtp_).

Finally, this study assessed the potential ecological risks of selected OP triesters and transformation products for organisms at various trophic levels (algae, crustaceans, and fish), and the results are shown in Figure 3. In general, the ecological risk of OP triesters in mangrove sediments in the Beibu Gulf was low. Algae seems more sensitive to the toxicity of TCEP, while crustaceans to TPHP toxicity. However, the ecological risks of the OP diesters were much higher than their parent compounds. For example, DPHP exhibited medium risk to organisms at most sampling sites, while DNBP showed high risk to algae and crustacea and medium risk to fish at almost all the sites of the studied mangrove in the Beibu Gulf. This is mainly due to the high concentrations of DPHP and DNBP detected in the studied mangrove sediment and their higher toxicity. Nevertheless, experimental research data on the toxicity of organophosphate metabolites to common aquatic organisms is still unknown. Therefore, the predictive toxicity data from the Estimation Program Interface (EPI) Suite (Version 4.1) were employed to estimate their ecological risks in this study. Recently, one study revealed that DPHP could impact the cardiac development of zebrafish [53]. DPHP was also reported to exhibit cytotoxicity to chicken embryonic hepatocytes and to strongly alter the gene expression [54]. We hypothesize that DPHP possibly has similar toxicity potential to other aquatic organisms. Based on these results, special attention should be paid to the occurrence of OP transformation products in mangrove ecosystems in the Beibu Gulf, particularly DNBP and DPHP. And further research on related pollution sources is needed in order to prevent more contamination and ecological threats to aquatic organisms. Additionally, although the ecological risks of OP triesters were low, given the mixed use of multiple OP triesters in industries and daily lives, as well as the coexistence of OP triesters and transformation products in the environment, the total HQ values of all the selected OPEs should not be neglected. To the best of our knowledge, this is the first report about the risk assessment of OP transformation products in a mangrove ecosystem.

## 3. Materials and Methods

### 3.1. Studied Areas and Sample Collection

**Figure 4:**
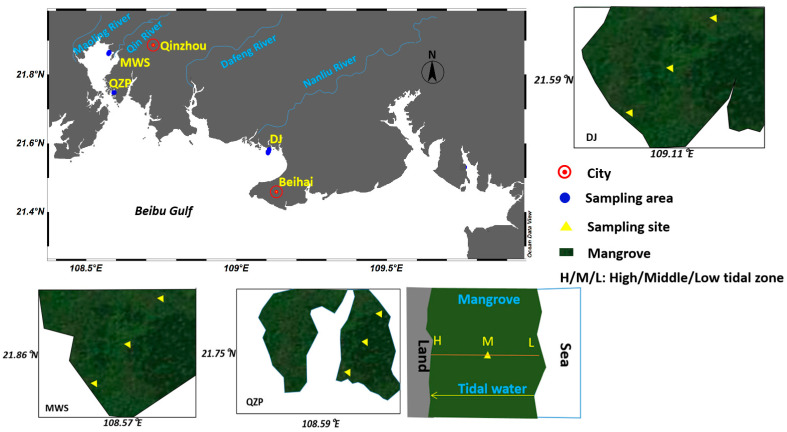
Sampling stations for surface sediments in three mangrove wetlands.

Surface sediment samples were collected and analyzed in three mangrove forests in the northern Beibu Gulf, South China Sea in October 2020, and detailed information on the selected sampling locations is provided in Figure 4 and Table S2. The Maowei Sea (MWS) mangrove wetland is located around the estuaries of the Qinjiang River, Mao Lingjiang River and Da Lanjiang River. Qinzhou Port (QZP) mangrove is close to the Qinzhou Port and also near a scenic area. Both the MWS and QZP mangrove wetlands belong to Qinzhou City, Guangxi Province, while the Dangjiang River (DJ) mangrove is located in rural areas of the Beihai city. 

An inter-tidal section was selected in a direction vertical to the coast for every sampling area, and the sampling sites were set at the high, middle and low tidal zones (namely, in the direction from land to sea) of the mangrove forests. The distances (away from the land) among the three sampling locations in every section were 33–763 m, which depended on the scale of the selected mangrove forest. A GPS was used to record the locations during the sample collection. Surface sediment samples (0–5 cm) were collected with a stainless-steel grab sampler during the lowest tidal period, and two separate quadrats (1.5 m × 1.5 m) were set as replicates at each sampling site. Together, n = 18 (9 sites, and 2 replicates per site) sediment samples were obtained and then transported immediately on ice to the laboratory and stored at −20 °C before analysis. 

### 3.2. Chemical and Reagents

Hexane, dichloromethane, acetonitrile (ACN) and methanol (all HPLC grade) were purchased from Merck (Darmstadt, Germany). LC-MS-grade ammonium acetate and formic acid were purchased from Sigma-Aldrich (St. Louis, MD, USA). Ultra-pure water (18.2 MΩ) cm was produced for a Milli-Q Advantage A10 system (Millipore Corporation, Bedford, MA, USA). 

N = 12 OP triesters, including TBOEP, TPHP, triethyl phosphate (TEP), 2-ethylhexyl diphenyl phosphate (EHDPP), tri-n-butyl phosphate (TNBP), tripropylphosphate (TPRP), tris(2-ethylhexyl) phosphate (TEHP), tri-iso-butyl phosphate (TIBP), tris(methylphenyl) phosphate (TMPP), tris(2-chloroethyl) phosphate (TCEP), tris(1,3-dichloropropan-2-propyl) phosphate (TDCIPP), and tris(2-chloropropyl) phosphate (TCIPP) were purchased from Dr. Ehrenstorfer GmbH (Oakville, ON, Canada) and AccuStandard (New Haven, CT, USA). N = 5 OP diesters, including bis(2-chloroisopropyl) phosphate (BCIPP), di-n-butyl phosphate (DNBP), DPHP, BBOEP, and bis(1,3-dichloroisopropyl) phosphate (BDCIPP), as well as n = 2 OH-OPEs, including BBOEHEP and 3-OH-TBOEP, were purchased from Toronto Research Chemicals (North York, ON, Canada). The purity of the above purchased standards was >94%. Related isotopically labeled OPEs were also purchased as internal standards from Cambridge Isotope Laboratories (Andover, MA, USA). 

### 3.3. Sample Pretreatment and Instrumental Analysis

The sample pretreatment and analysis procedures for the selected OP triesters, diesters and OH-OPEs in the sediments followed our previous method [3]. Briefly, for the OP triesters, 3-OH-TBOEP and BBOEHEP, 2 g of mixed and dry sediment sample was spiked with 20 ng isotopically labeled standards, and then the ultrasonic extraction was performed three times with ACN solvent, after which the extract was centrifuged, combined and purified with a pre-conditioned ENVI-C18 cartridge (6 mL, 500 mg; SUPELCO). Finally, the eluent was reconstituted in methanol before analysis. For the OP diesters, similar procedures to those for the OP triesters were conducted for the extraction, purification and concentration processes, except the extraction and elution solvent using methanol and the SPE cartridge used Oasis HLB cartridges (6 mL, 500 mg; Waters) instead.

An Agilent 1290 Infinity II LC system coupled to a 6470 triple quadrupole MS (Agilent Technologies, Mississauga, ON, Canada) was used for the compound analysis. The twelve OP triesters, BBOEHEP and 3-OH-TBOEP were analyzed in the ESI^+^ mode, while the OP diesters in the ESI^−^ mode. A 5 mmol/L ammonium acetate (0.1% formic acid) and pure water (0.1% formic acid) were used as the aquatic phase in the ESI+ mode and the ESI^−^ mode, respectively. And methanol was applied as the organic phase for the separation of all the targets. Detailed information on the chromatographic separation and MS operational conditions is provided in the Supplementary Information. The total organic carbon (TOC) contents of the mangrove sediments were analyzed using a Solid TOC Analyzer (TOC-L CPH, Shimadzu, Japan), and the details were introduced in our previous report [1]. The detailed LC-MS/MS parameters and sample pretreatment procedures are listed in Texts S1, S2 and Table S3. 

### 3.4. Ecological Risk Assessment of OPEs 

The ecological risk assessment followed our previous report [55,56]. The hazard quotient (HQ) method based on Equation (1) was calculated to evaluate the potential ecological risk of OPEs in mangroves in the Beibu Gulf.

HQ = MEC_sed_/PNEC_sed_ (1)

where MEC_sed_ is the measured environmental concentration (ng/g dw); and PNEC_sed_ is the predicted no effect concentration in the sediment (ng/g dw), which is derived by equilibrium partitioning between the water and sediment, see Equations (2) and (3):

PENC_sed_ = Koc·*f*_oc_·PENC_aqua_ (2)

PENC_aqua_ = L(E)C_50_/AF (3)

a550 where *f*_oc_ is the percentage of organic carbon in the sediment, and Koc is the organic carbon–water partition coefficient (L/kg). The PENC_aqua_ was calculated with the 50% lethal/effect concentration (LC50/EC50) of each target and the assessment factor (AF = 1000) [57,58,59,60,61]. The LC50/EC50 and PENC values are listed in Table S4.

A result of 0.01 ≤ HQ < 0.1 indicates low risk; 0.1 ≤ HQ < 1 indicates moderate risk; and HQ ≥ 1 indicates the high risk and special attention should be paid. 

### 3.5. Quality Assurance and Quality Control

All the glassware used in the extraction and cleanup processes was treated at 450 °C for four hours to remove any possible organic contamination. After cooling down to room temperature, all the glassware was covered with aluminum foil and rinsed with hexane, dichloromethane and ACN in sequence before use. To ensure the quality of the OPE measurements, eight-point calibration curve was employed for the OPE quantification, and procedural blank analysis was performed before each batch of samples (n = 12) to check for background contamination. Overall, each target exhibited excellent linearity with r^2^ > 0.99, and relatively low levels of background contamination (0.1–0.82 ng/g dw) were identified for some targets in the procedural blanks, with the exception of DNBP (1.34 ng/g dw), which is also one of the most commonly used plasticizers. Triplicate sample analyses were performed to verify the consistency of the results, and the average concentration after background subtraction was calculated as the final value. The limit of quantification (LOQ) was defined as ten times the signal-to-noise ratio for compounds not detected in the blank samples; for others, the LOQ was defined as the mean value of the target compound detected in the blanks plus thrice the standard deviation [3,56]. The LOQs of all the targets ranged from 0.009–1.96 ng/g dw (except DNBP 5.00 ng/g dw) in the sediment samples. The spiked recoveries at 10 ng/g ranged from 68~137%. Detailed information on the LC-MS/MS parameters, blank contamination, LOQs and recoveries is shown in Table S3.

### 3.6. Data Analysis

The chromatographic data were analyzed using an Agilent MSD ChemStation. Statistical analysis was performed with Microsoft^®^ Office Excel 2007, IBM^®^ SPSS^®^ Statistics 26 (Pearson’s correlation analysis), and Origin^®^ 8.5. Statistical significance at 95% confidence (*p* < 0.05) was accepted throughout this study. The sampling sites and concentration distribution graphs were generated using Ocean Data View (version: 5.3.0).

## 4. Conclusions

In this paper, the occurrence, distribution and ecological risk assessment of OPEs, including both the parent OP triesters and the transformation products, were investigated for the first time in mangrove wetlands. The results showed that OPEs widely existed in the mangrove sediments in the Beibu Gulf, South China Sea. Overall, TCIPP, TCEP, TPHP and TNBP were the main components of the OP triesters, while DNBP and DPHP were the main components of the OP transformation products among all the sampling areas. The levels of the Σ_12_OP_tri_ concentration in the MWS were much higher than in the other two mangrove areas; however, the results were just the contrary for the total amount of selected OP transformation products. The Pearson correlation was analyzed between the OPE concentrations and TOC contents, as well as between the OP triesters and their transformation products. The results showed that the TOC and degradation are not the main factors influencing the OPEs’ distribution in mangrove sediments. Finally, the ecological risks of OPEs were also accessed, and the results indicated special attention should be paid to OP transformation products in the mangrove wetlands of Beibu Gulf, especially DNBP and DPHP. Future studies should operate constant monitoring and expand the sampling range of mangrove areas to cover different functional zones, and simultaneously collect samples of both mangrove biota and plants for comprehensive analysis. Meanwhile, the toxicity of OP diesters in different aquatic organisms needs further study in order to more accurately assess their ecological risks. Finally, the local government authorities should formulate pollution control measures for OPEs and implement them promptly.

## Figures and Tables

**Table 1:** Concentrations of organophosphate triesters and their transformation products in mangrove sediments in the Beibu Gulf (ng/g dw).

Stations	MWS	QZP	DJ	Mean	DF/%
**Organophosphate triesters**					
TEP	n.d.	n.d.	n.d.	n.d.	0
TCEP	2.27–7.58	<LOQ	<LOQ	1.57	100
TCIPP	2.90–18.45	2.04–2.48	2.69–6.10	4.87	100
TPRP	n.d.	n.d.	n.d.	n.d.	0
TDCIPP	0.18–0.28	0.10–0.14	0.06–0.08	0.14	100
TPHP	0.44–6.86	2.25–3.53	0.11–0.16	2.40	100
TIBP	<LOQ	<LOQ	<LOQ	<LOQ	100
TNBP	2.21–2.51	1.69–3.55	0.99–2.72	2.19	100
TBOEP	0.03–0.06	0.02	0.02–0.03	0.03	100
TMPP	0.14–0.52	0.02–0.04	0.36–2.68	0.59	100
EHDPP	<LOQ–0.79	<LOQ	<LOQ	0.19	100
TEHP	2.54–5.27	<LOQ–0.74	0.35–0.63	1.75	100
**Sum**	**15.09–39.96**	**7.48–10.46**	**6.43–10.98**	**14.1**	**/**
**Transformation products**					
BCIPP	<LOQ	<LOQ	<LOQ	<LOQ	100
DPHP	<LOQ–1.17	0.66–0.93	0.43–1.90	0.84	100
DNBP	2.47–15.71	16.5–21.4	17.17–19.45	16.38	100
BDCIPP	n.d.	n.d.	n.d.–<LOQ	<LOQ	11
BBOEP	n.d.	n.d.–<LOQ	n.d.	<LOQ	22
BBOEHP	<LOQ	<LOQ	<LOQ	<LOQ	100
3-OH-TBOEP	n.d.– < LOQ	n.d.– < LOQ	n.d.– < LOQ	<LOQ	33
**Sum**	**3.33–17.57**	**17.76–22.50**	**17.78–21.57**	**17.44**	**/**

Note: “n.d.” means not detected; “<LOQ” means the amount was lower than LOQ, “DF” means detection frequency; “MWS” means Maowei Sea mangrove area; “QZP” means Qinzhou Port mangrove area; “DJ” means Dangjiang mangrove area.

## Data Availability

All data are contained within the article.

## Supplementary Materials

The following supporting information can be downloaded at https://www.mdpi.com/article/10.3390/molecules29030736/s1, Text S1: Sample pretreatment; Text S2: Instrumental analysis; Table S1: Results of the Pearson correlation analysis between the OPE concentrations and the TOC contents; Table S2: Investigation of sampling sites in mangrove wetlands in the Beibu Gulf, South China Sea; Table S3: Predicted no effect concentrations (PNEC) of OPEs based on EC50 or LC50; Table S3: The LC-MS/MS parameters, blank contamination, LOQs and recoveries of OP triesters and their degradation products; Table S4: Predicted no effect concentrations (PNEC) of OPEs based on EC50 or LC50.
