# Supplementary material for: Organophosphate Triesters and Their Transformation Products in Sediments of Mangrove Wetlands in the Beibu Gulf, South China Sea
Source: Molecules. 2024 Feb 5;29(3):736. doi: 10.3390/molecules29030736 (PMC10856239; doi:10.3390/molecules29030736)
Supplement: Supplementary file 1 [file molecules-29-00736-s001.zip › molecules-2703822-supplementary.pdf]

Supplementary Information

Organophosphate Triesters and Their Transformation Products in Sediments of Mangrove Wetlands in the Beibu Gulf, South China Sea

Li Zhang [1,2], Yongze Xing [1,2,*], Peng Zhang [3], Xin Luo [4] and Zengyuan Niu [4]

[1] Guangxi Key Laboratory of Beibu Gulf Marine Resources, Environment and Sustainable Development, Fourth Institute of Oceanography, Ministry of Natural Resources, Beihai 536000, China; zhangli@4io.org.cn
[2] Key Laboratory of Tropical Marine Ecosystem and Bioresource, Fourth Institute of Oceanography, Ministry of Natural Resources, Beihai 536000, China
[3] College of Chemistry and Environmental Science, Guangdong Ocean University, Zhanjiang 524088, China; zhangpeng@gdou.edu.cn
[4] Technology Center of Qingdao Customs District, Qingdao 266109, China; rossi612@hotmail.com (X.L.); zyniuqd@163.com (Z.N.)
* Correspondence: Correspondence: haiyangsisuo2020@163.com

Text S1 Sample pretreatment

Prior to chemical analysis, sediment samples were freeze-dried by CHRIST Alpha 1-4 LSCbasic (Osterode, Germany), then grounded using Retsch MM400 (Haan, Germany), and homogenized by sieving through a stainless steel 80-mesh sieve.

For all OP triesters and two hydroxylated degradation products (BBOEHEP and 3-OH-BBOEP), 2 g of the homogenized sediment was added in a 30 mL glass tube, after which 20 ng isotopically labeled standards of TNBP-d27, TPHP-d15, TCIPP-d18 and BBOEHEP-d4 were added as internal standards (IS), and then extracted by an ultrasonic generator (40 KHz, KQ-500DE, Kun Shan, China) with 10 mL of acetonitrile at room temperature for 15 min. Then the extract was centrifuged for 15 min at 3500 rpm at 4 ˚C to collect the supernatant. Above process repeated twice. The extracts were combined and reduced to 1–2 mL under a nitrogen stream at room temperature. Forty ml of water was added and the extracts were loaded onto a pre-conditioned (5 ml methanol) and pre-equilibrated (5 ml ultrapure water) ENVI-C18 column (6 mL, 500 mg; SUPELCO), with a flow rate of 5–10 mL/min. After loading, the sorbent was washed with 5 mL of ultrapure water and dried under vacuum for 30 min. Following that, 9 mL acetonitrile (3 mL each time) was used to elute the targets retained on the cartridge, and then the eluent was condensed to near dryness under a gentle nitrogen flow at room temperature. Finally, the sample was reconstituted in 200 μL methanol and and filtered by a 0.22 μm PTFE membrane before analysis.

For OP diesters, 2 g of the homogenized sediment was added in a 30 mL glass tube, after which 50 ng of DPHP-d10 and BDCIPP-d10 were spiked as IS, and then extracted by UAE with 10 mL of methanol at ambient temperature for 15 min. Then the extract was centrifuged for 10 min at 3500 rpm at 4 °C to collect the supernatant. Above process repeated twice. The extracts were combined and reduced to 1–2 mL under a nitrogen stream at room temperature. Forty ml of water was added and the extract was purified by SPE on Oasis HLB cartridges (6 mL, 500 mg; Waters) according to the OP tiesters extraction protocol, except that the elute solution used methanol as instead. The eluent was then evaporated to near dryness. Finally the sample was reconstituted in 200 μL methanol and filtered by a 0.22 μm PTFE membrane before analysis.

Text S2 Instrumental analysis

Analysis was performed with an Agilent 1290Ⅱ LC system coupled to a 6470 MS (Agilent Technologies, Mississauga, Canada). Chromatographic separation of targets was performed in an Agilent ZORBAX C18 column (4.6 mm × 100 mm, 1.8 μm). The injection volume was 5 μL, and the column temperature was 40 °C. Twelve OP triesters and their two hydroxylated degradation products were analyzed in the ESI^+ mode, using 5 mmol/L ammonium acetate (containing 0.1% formic acid (FA)) (A)-methanol (B) as mobile phase. OP diesters were analyzed in the ESI^- mode, using water (containing 0.1% FA) (A)-methanol (B) as mobile phase. The LC gradient elution program under ESI^+ condition was as follows: 0–7 min, 60-80% B, 7–16 min, 97% B, 20 min, 97% B, 20.1 min, 60%, 20.1–24 min, 60% B. The LC elution gradient program under ESI^+ condition was as follows: 0–7 min, 50–95% B, 7–10 min, 95% B, 10.1 min, 50% B, 10.10–15 min, 50% B. The total flow rate was 0.3 mL/ min.

For the triple-quadrupole MS, the operational conditions were as follows: gas temperature 250 °C; gas flow 9 L/min; sheath gas temperature 350 °C; and sheath gas flow 11 L/min. Capillary in positive and negative ionization mode was 4 kV and 3.5 kV, respectively, while nozzle voltage was 500 V in both ionization modes. Chromatograms were recorded using multiple reactions monitoring (MRM) mode. Nitrogen was applied as desolvation and collision gas.

Table S1. Results of Pearson correlation analysis between the OPE concentrations and the TOC contents.

	OP triesters		OP transformation products	
Sampling site	P	r^2	P	r^2
MWS	1.000 *	0.018	-0.993	0.075
QZP	0.516	0.655	-0.977	0.135
DJ	0.533	0.642	-0.636	0.561

Note: "*" means correlation is significant ($p < 0.05$);

Table S2. Investigation of sampling sites in mangrove wetlands in Norther Beibu Gulf, South China Sea.

Collecting station	abbreviation	longitude/◦	latitude/◦
Dangjiang Mangrove Area - Low tide zone	DJ–L	109.1048	21.5728
DangjiangMangrove Area - Middle tide zone	DJ–M	109.1068	21.5765
Dangjiang Mangrove Are a- High tide zone	DJ–H	109.1091	21.5820
Maowei Sea Mangrove Area - Low tide zone	MWS–L	108.5741	21.8620
Maowei Sea Mangrove Area - Middle tide zone	MWS–M	108.5756	21.8636
Maowei Sea Mangrove Area - High tide zone	MWS–H	108.5773	21.8656
Qinzhou Port Mangrove Area - Low tide zone	QZP–L	108.5931	21.7483
Qinzhou Port Mangrove Area - Middle tide zone	QZP–M	108.5923	21.7480
Qinzhou Port Mangrove Area - High tide zone	QZP–H	108.5915	21.7479

Table S3. The LC-MS/MS parameters, blank contamination, LOQs and recoveries of OP triesters and their degradation products.

No.	Compound	Retention time/min	Quantification transition (m/z)	Blank contamination （ng/g）	LOQ （ng/g）	Spiked recovery/%, (RSD)
**Organophosphate triesters**						
1	TEP	5.76	183>99<br>183>81	0.10	0.10	137（2.36）
2	TCEP	6.54	285>63<br>285>161	0.66	0.70	81（15.57）
3	TCIPP	10.01	327>175<br>327>99	0.82	0.95	68（3.40）
4	TPRP	10.10	225>99<br>225>81	n.d.	1.16	110（3.07）
5	TDCIPP	12.51	431>99<br>431>208	n.d.	0.032	71（2.07）
6	TPHP	12.64	327>77<br>327>152	n.d.	0.0090	130（2.81）
7	TIBP	14.14	267>99<br>267>155	0.81	0.82	134（1.10）
8	TNBP	14.38	267>99<br>267>155	0.28	0.37	119（10.17）
9	TBOEP	15.27	399>199<br>399>299	n.d.	0.018	125（2.33）
10	TMPP	16.56	369>165<br>369>91	n.d.	0.021	106（2.12）
11	EHDPP	17.56	363>251<br>363>77	n.d.	0.52	102（6.39）
12	TEHP	21.76	435>99	n.d.	0.33	76（7.48）

No.	Compound	Retention time/min	Quantification transition (m/z)	Blank contamination （ng/g）	LOQ （ng/g）	Spiked recovery/%, (RSD)
			435>113			
**Organophosphate hydroxylated degradation products**						
13	BBOEHEP	9.73	343>243<br>343>45	0.20	0.35	99 (6.03)
14	3-OH-TBOEP	11.14	415>101<br>415>57	n.d.	0.07	86 (4.46)
**Organophosphate diesters**						
15	BCIPP	6.50	249>35<br>251>37	n.d.	5.00	104 (11.82）
16	DPHP	6.81	249>93<br>249>155	0.37	0.39	85 (6.39)
17	DNBP	8.18	209>153<br>209>137	1.34	1.96	80 (7.96)
18	BDCIPP	8.39	319>35<br>317>35	n.d.	0.50	84 (4.75)
19	BBOEP	9.22	297>79<br>297>197	0.21	0.24	104 (10.05)

Table S4. Predicted no effect concentrations (PNEC) of OPEs based on EC50 or LC50.

Compound	Organism	Exposure duration	L(E)C_{50} (mg/L)	logK_{oc}	$PNEC_{aqua}$ (μg/L)	$PNEC_{sed}$ (ng/g)	Ref.
TCEP	Algae *(Scenedesmus subspicatus)*	96h	1.2	2.48	1.2	36.24	[1]
	Crustacea *(Daphnia magna)*	24h	235	2.48	235	7096.89	
	Pisces (Carassius auratus)	96h	90	2.48	90	2717.96	
TDCIPP	Algae *(Pseudokirchneriella subcapitata)*	96h	12	2.35	12	268.65	[1]
	Crustacea *(Daphnia magna)*	48h	3.8	2.35	3.8	85.07	
	Pisces (Carassius auratus)	96h	5.1	2.35	5.1	114.17	
TCIPP	Algae *(Pseudokirchneriella subcapitata)*	96h	47	2.71	47	2410.45	[1]
	Crustacea *(Daphnia magna)*	48h	63	2.71	63	3231.03	
	Pisces (Brachydanio rerio)	96h	56	2.71	56	2872.02	
TPHP	Algae *(Pseudokirchneriella subcapitata)*	96h	2	3.72	2	1049.61	[1]
	Crustacea *(Mysidopsis bahia)*	96h	0.25	3.72	0.25	131.20	
	Pisces (Cyprinodon variegatus)	96h	0.44	3.72	0.44	230.92	
TNBP	Algae *(Pseudokirchneriella subcapitata)*	96h	4.4	3.28	4.4	838.40	[1]
	Crustacea *(Daphnia magna)*	24h	35	3.28	35	6669.11	
	Pisces (Brachydanio rerio)	96h	11.4	3.28	11.4	2172.23	
TIBP	Algae *(Scenedesmus subspicatus)*	96h	30	3.6	30	11943.22	[1]
	Crustacea *(Daphnia magna)*	48h	11	3.6	11	4379.18	
	Pisces (Oncorhynchus mykiss)	96h	23	3.6	23	9156.46	
TBOEP	Algae *(Scenedesmus quadricauda)*	96h	0.3	3.65	0.3	134.01	[2]
	Crustacea *(Daphnia magna)*	48h	75	3.65	75	33501.27	[1]
	Pisces (Pimephales promelas)	96h	11.2	3.65	11.2	5002.86	
TEHP	Algae *(Scenedesmus subspicatus)*	72h	34	6.87	34	25204548.20	[1]
	Pisces (*Oryzias latipes*)	48h	500	6.87	500	370655120.65	
	Crustacea *(Daphnia magna)*	48h	11	6.87	11	8154412.65	[2]
EHDPP	Crustacea	/	/	6.87	0.015	29.19	[3]
TEP	Algae *(Scenedesmus subspicatus)*	72h	900.8	1.68	900.8	4311.50	[1]
	Crustacea (*Nitroca spinipes*)	96h	950	1.68	950	4546.99	

TMPP	Pisces (*Alburnus alburnus*)	96h	2350	1.68	2350	11247.81	[2]
	Algae (*Ankistrodesmus falcatus*)	96h	1.5	4.35	1.5	3358.08	
	Crustacea (*Daphnia magna*)	48h	0.27	4.35	0.27	604.45	
TPrP	Pisces (Zebrafish)	96h	252	2.83	252	17037.29	[4]
3-OH-TBOEP	/	/	/	/	/	/	/
BBOEHEP	/	/	/	/	/	/	/
BCIPP	Daphnid	48h	225.77	0.00	225.77	22.58	a
	Green Algae	96h	148.14	0.00	148.14	14.81	a
	Fish(SW)	96h	515.15	0.00	515.15	51.51	a
DPHP	Daphnid	48h	20.63	2.08	20.63	4.29	a
	Green Algae	96h	21.48	2.08	21.48	4.47	a
	Fish(SW)	96h	42.41	2.08	42.41	8.82	a
DNBP	Daphnid	48h	55.41	2.18	55.41	12.08	a
	Green Algae	96h	46.09	2.18	46.09	10.05	a
	Fish(SW)	96h	119.83	2.18	119.83	26.12	a
BDCIPP	Daphnid	48h	105.47	2.64	105.47	27.84	a
	Green Algae	96h	84.01	2.64	84.01	22.18	a
	Fish(SW)	96h	230.32	2.64	230.32	60.80	a
BBOEP	Daphnid	48h	419.47	3.78	419.47	158.56	a
	Green Algae	96h	156.85	3.78	156.85	59.29	a
	Fish(SW)	96h	527.26	3.78	527.26	199.30	a

[a] Estimated by Estimation Program Interface (EPI) Suite, V 4.1; U.S. EPA 13.

"/" means related data can not be obtained.

References

1. Verbruggen, E.M.J., Rila, J.P., Traas, T.P., Posthuma-Doodeman, C.J.A.M., Posthumus, R. Environmental Risk Limits for several phosphate esters, with possible application as flame retardant. RIVM report. 2005.
2. Liu, Y., Gong, S., Ye, L., Li, J., Liu, C., Chen, D., Fang, M., Letcher, R.J., Su, G. Organophosphate (OP) diesters and a review of sources, chemical properties, environmental occurrence, adverse effects, and future directions. Environ. Int. 2021 155, 106691.
3. Chen, M.; Gan, Z.; Qu, B.; Chen, S.; Dai, Y.; Bao, X. Temporal and seasonal variation and ecological risk evaluation of flame retardants in seawater and sediments from Bohai Bay near Tianjin, China during 2014 to 2017. Mar. Pollut. Bull. 2019, 146, 874 – 883.
4. Wang, X., Zhu, Q., Yan, X., Wang, Y., Liao, C., Jiang, G. A review of organophosphate flame retardants and plasticizers in the environment: Analysis, occurrence and risk assessment. Sci. Total Environ. 2020. 731, 139071.
